# A Novel Serine Protease Inhibitor PE-BBI Ameliorates Cockroach Extract-Mediated Airway Epithelial Barrier Dysfunction

**DOI:** 10.3390/biom10040515

**Published:** 2020-03-28

**Authors:** James A. Reihill, Xuan Ouyang, Zhixuan Yang, Lisa E. J. Douglas, Mei Zhou, Tianbao Chen, S. Lorraine Martin

**Affiliations:** School of Pharmacy, Queen’s University Belfast, Belfast BT9 7BL, Northern Ireland, UK; j.reihill@qub.ac.uk (J.A.R.); xouyang01@qub.ac.uk (X.O.); zyang09@qub.ac.uk (Z.Y.); l.douglas@qub.ac.uk (L.E.J.D.); m.zhou@qub.ac.uk (M.Z.); t.chen@qub.ac.uk (T.C.)

**Keywords:** cockroach, allergen, protease, protease inhibitor, airway epithelium, airway epithelial barrier, airway epithelial barrier dysfunction

## Abstract

Epithelial barrier dysfunction, characteristic of allergic airway disease may be, at least in part, due to the action of allergen-associated protease activities. Cockroach allergy is a major global health issue, with cockroaches containing considerable serine trypsin-like protease (TLP) activity. The present study sought to evaluate two novel protease inhibitors (PE-BBI and pLR-HL), recently isolated from amphibian skin secretions, for their potential to neutralise cockroach TLP activity and to determine any protective effect on cockroach-induced airway epithelial barrier disruption. Inhibitor potencies against the cockroach-associated activities were determined using a fluorogenic peptide substrate-based activity assay. 16HBE14o^-^ cells (16HBE; a bronchial epithelial cell line) were treated with cockroach extract (CRE) in the presence or absence of the compounds in order to assess cell viability (RealTime Glo luminescent assay) and epithelial barrier disruption (transepithelial resistance and paracellular dextran flux). PE-BBI potently and selectively inhibited CRE TLP activity (pIC_50_ -8), but not host (16HBE) cell surface activity, which conferred protection of 16HBE cells from CRE-induced cell damage and barrier disruption. Novel protease inhibitor strategies such as PE-BBI may be useful for the treatment of allergic airway disease caused by cockroach proteases.

## 1. Introduction

Sensitization and exposure to allergens is a risk factor for allergic respiratory disease including asthma, which is a chronic condition affecting approximately 340 million people worldwide [1]. Allergic sensitisation results from complex interactions between the allergen and the host with the airway epithelium representing the first site of interaction [2]. The airway epithelium forms a crucial barrier at the interface between the host and the inhaled environment protecting against microorganisms, airborne irritants and allergens [3]. In allergic inflammatory disease, increased epithelial permeability is characteristic [3]. Aeroallergens and other co-factors (e.g., cigarette smoke, pollution) interact with epithelial innate immune receptors including Toll-like and protease-activated receptors, which results in elevated production of pro-inflammatory cytokines that drive T-helper 2 (Th2)-like adaptive immunity [3]. Disruption of the airway epithelial barrier enables allergens to interact with subepithelial dendritic cells that ultimately results in naïve T cells becoming Th2 cells [3].

Over the last 50 years, numerous studies have shown that environmental cockroach exposure can cause sensitization and is associated with asthma particularly in children and young adults in inner city urban environments [4]. Allergens from various sources have been widely shown to possess proteolytic activity that can affect airway epithelial barrier function and modulate allergic airway inflammation [5,6,7,8]. Cockroach serine protease activity disrupts the airway epithelial cell barrier by interfering with tight junctions that are necessary for forming tight cell–cell contacts [8,9], and moreover promotes airway hyper-responsiveness and mucin secretion [5,10]. Cockroach-derived protease-stimulated airway epithelial cells exhibit increased release of pro-inflammatory cytokines including IL-6, IL-8, TSLP and IL-33 in a protease-dependent manner [8,11,12,13].

We have previously reported two novel Bowman–Birk protease inhibitor-type peptides, namely PE-BBI [14] and pLR-HL [15], isolated from frog skin secretions (*Pelophylax esculentus* and *Hylarana latouchii* species, respectively). The Bowman–Birk inhibitor (BBI) family are typical potent serine protease inhibitors, which occur extensively in the seeds of leguminous and gramineous plants. According to their primary structural homologies, serine protease inhibitors can be classified into at least 10 families that include those possessing Kunitz, Kazal and Bowman–Birk motifs [16]. They function by combining with their cognate enzyme in a substrate-like manner, being mediated by the exposed reactive site loop which is complementary to the protein active site, and form a stable complex [16,17]. Recently, BBIs have attracted much interest—particularly due to their array of potential applications, which include defence against insects in transgenic plants and broader clinical applications such as the prevention of cancer, inflammatory and allergic disorders [18]. A drug formulation termed BBI concentrate (BBIC), a soya bean extract rich in BBIs, was granted investigational new drug status by the US Food and Drug Administration (FDA) in April 1992 [19] and showed indications of clinical efficacy at Phase 1 for both benign prostatic hyperplasia [20] and oral leucoplakia [21].

The main aim of the present study was to investigate whether the novel, natural bioactive serine protease inhibitors (PE-BBI and pLR-HL) possess efficacy against cockroach extract (CRE) trypsin-like protease (TLP) activity and, subsequently, to determine whether they play a protective role in regard to CRE-mediated airway epithelial cell damage. Here, we report PE-BBI to be a potent inhibitor of CRE TLP activity but not host airway TLP activity. PE-BBI ameliorated damage inflicted on airway epithelial cells on exposure to cockroach-associated proteases. 

## 2. Materials and Methods

### 2.1. Extract and Reagents 

Whole-body German cockroach extract was sourced from Greer Laboratories (USA). All other reagents were obtained from Sigma-Aldrich unless otherwise indicated. 

### 2.2. Peptide Inhibitors

All methodological details pertaining to the isolation and initial characterisation of both peptide inhibitors, PE-BBI and pLR-HL, have been reported in detail previously [14,15]. PE-BBI and pLR-HL were synthesised by GenScript (> 98% purity). 

### 2.3. Determination of Putative Protease Inhibitor Potency versus Recombinant Trypsin and CRE TLP Activity

Synthetic replicates of PE-BBI and pLR-HL, as well as the small-molecule compound gabexate mesylate (GM; Tocris), were serially quantified to assess both trypsin or CRE protease inhibitory activity in the range of 0.01–10 μM. Cleavage of the fluorogenic peptide substrate Boc-QAR-NH_2_Mec (50 µM final concentration) (R&D Systems) was used to assay TLP activity with the rate of substrate hydrolysis continuously recorded at λ_ex_ 380 nm and λ_em_ 460 nm using a FLUOstar Optima microplate reader (BMG Labtech). All inhibition assays were performed in microtitre plates maintained at 37 °C in a final volume of 100 μL. Curve fitting and pIC_50_ (-logIC_50_) determinations were carried out by fitting to a four-parameter logistic equation (GraphPad Prism).

### 2.4. Cell Culture

Studies were performed using a SV-40-transformed human bronchial epithelial cell line (16HBE14o^-^) [22]. The 16HBE cells were grown on collagen-coated T75 flasks (Corning) using bronchial epithelial growth medium (BEGM) (Lonza), in a humidified cell culture incubator supplemented with 5% CO_2_. After trypsinization, cells (5 × 10^5^ cells/cm^2^) were seeded onto semipermeable transwell filters (Corning) in BEGM and allowed to grow at liquid–liquid interface for 2 days then switched to DMEM/F-12 medium supplemented with 2% (*v*/*v*) Ultroser G (Pall-BioSpera) for a further 2 days (liquid–liquid interface), at which point cultures were exposed to air–liquid interface conditions for a further 5–7 day period, whereupon experiments were conducted.

### 2.5. The 16HBE Cell Surface TLP Activity Assay

Polarised 16HBE cells grown on transwell semi-permeable filters were used to assess cell surface TLP activity. Apical cell-surface (membrane-attached) proteolytic activity was measured in stringently washed (using PBS) cells via direct addition of a fluorogenic peptide substrate (50 µM Boc-QAR-–NH_2_Mec) to the apical compartment with continuous measurement of the formation of –NH_2_Mec (at λ_ex_ 380 nm and λ_em_ 460 nm) over a 1 h period. Surface activity was determined in the presence and absence of protease inhibitors (GM, pLR-HL or PE-BBI) which were added at a final concentration of 50 µM. 

### 2.6. Cell Viability Assay

Cell viability was measured in submerged 16HBE cells using the non-lytic and homogeneous RealTime-Glo MT Cell Viability Assay (Promega) as per the manufacturer’s instructions. The 16HBE cells (2.5 × 10^4^ cells/well) were seeded in 96 well microplates for 24 hours prior to the addition of RealTime-Glo reagent and 50 µM test compound (protease inhibitors). CRE (16.5 µg protein/well) was then added and the luminescent signal (produced via the action of viable cells only) was measured 24 hours later using a Cytation 7 Cell Imaging Multi-mode Reader (Biotek) in order to quantify cell viability. 

### 2.7. Airway Epithelial Cell Barrier Measurement

Polarised 16HBE cells were treated apically with CRE in the presence or absence of the candidate protease inhibitor (50 µM) for 24 hours, and then transepithelial resistance was measured using the transepithelial current clamp (TECC)-24 system (EP devices). Paracellular flux of apically applied 10 kDa Texas red dextran (0.1 mg/mL) was monitored by recovering basolateral media 2 hours post treatment and measuring the fluorescent signal.

### 2.8. Statistical Analysis

Data are presented as the mean (SEM). Statistical differences between two groups were determined with the Mann–Whitney test, whereas differences between more than two groups were determined using Kruskal–Wallis analysis with post hoc Dunn’s test. All statistical tests were performed using GraphPad Prism software (version 8). P values of less than 0.05 were considered significant. 

## 3. Results

### 3.1. Evaluation of Putative Protease Inhibitors versus Trypsin Activities

**Figure 1:**
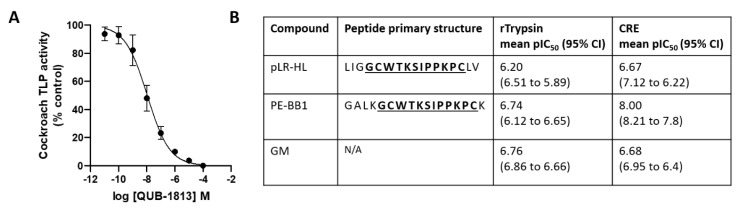
(**A**) Exemplar concentration–response (inhibition) curve analysis where the x-axis is the log QUB-1813 concentration (molar) and the y-axis the response (expressed as percentage inhibition calculated against the vehicle control value) for cockroach extract (CRE) trypsin-like protease (TLP) activity. (**B**) Summary table detailing the potency of candidate protease inhibitors tested against recombinant trypsin activity and the TLP activity present in CRE as assessed by fluorogenic peptide substrate-based assay. n ≥ 4.

Our two recently identified compounds, pLR-HL and PE-BBI, were examined against recombinant trypsin activity and compared with the commercially available serine protease inhibitor GM. In inhibition-response curve assays, all inhibitors were found to reduce trypsin activity in a dose-dependent manner, with similar pIC50 values obtained: pLR-HL (6.20), PE-BBI 6.74) and GM (6.76) (Figure 1). We next examined the impact of these compounds on CRE TLP activity, finding that pLR-HL and GM were similarly effective, with reasonably modest pIC50 values of ~6.7 evident for both (Figure 1). In comparison, PE-BBI was considerably more potent than either pLR-HL or GM, with a pIC50 value of 8.0 observed (Figure 1). 

### 3.2. Evaluation of Putative Protease Inhibitors versus 16HBE Cell Surface Activity

**Figure 2:**
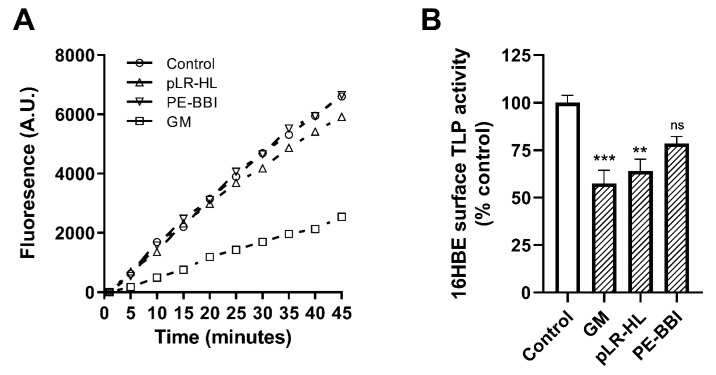
Evaluation of protease inhibitors compounds on polarised 16HBE surface TLP activity. (**A**) Typical kinetic trace demonstrating TLP cell surface protease activity in the presence or absence of putative inhibitor compounds. Summary data are quantified in panel (**B**). Data are presented as the mean ± SEMs (n = 7). ** P < 0.01, *** P < 0.001, ns (not significant).

We sought to evaluate whether PE-BBI or pLR-HL would impact host protease activity. TLP activity at the cell surface of polarised 16HBE cells was significantly reduced by the broad-spectrum inhibitor GM (*p* < 0.001) and pLR-HL (*p* < 0.001) but not PE-BBI (Figure 2). 

### 3.3. PE-BBI Protects 16HBE Cells from CRE-Induced Damage

**Figure 3:**
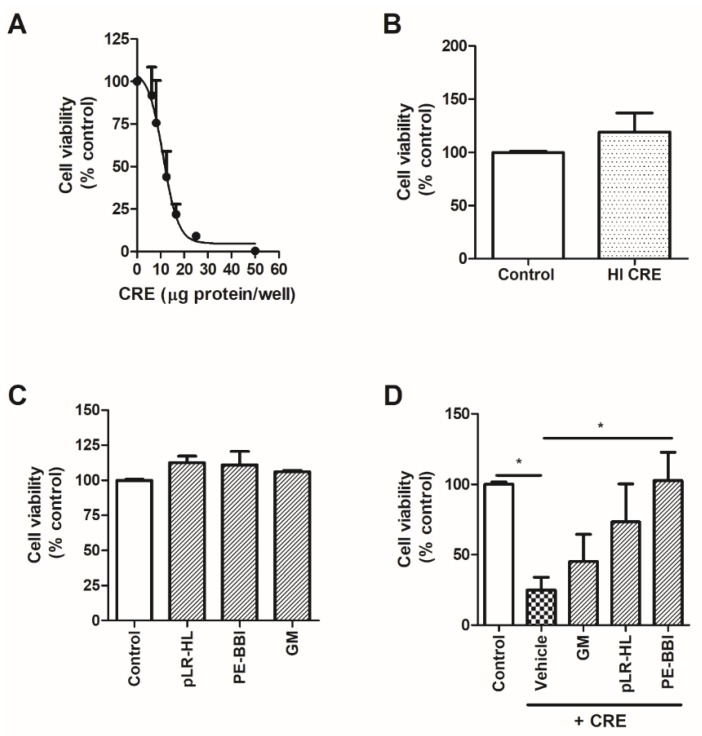
The effect of protease inhibitors on cockroach-mediated cell death (submerged 16HBE cells). (**A**) Addition of cockroach extract resulted in reduced cell viability in a dose-dependent manner. (**B**) Heat-inactivated cockroach extract (75 °C, 3 min), which completely abolishes proteolytic activity, does not affect cell viability when added at a high dose (50 µg/well). (**C**) Cell viability was unaltered by protease inhibitors added alone (at a 50 µM dose for 24 hr). (**D**) Cockroach extract (16.5 µg/well, 24 h) significantly elicited decreased cell viability that was rescued by co-administration of PE-BBI but not pLR-HL or GM (all added at a 50 µM dose). Data are presented as the mean ± SEMs (n = 6). * P < 0.05.

A previous study reported that GM ameliorates CRE-induced airway epithelial cell damage (BEAS-2B cells) [13]. Similarly, we demonstrate that CRE treatment reduced 16HBE cell viability in a dose-dependent manner (Figure 3A). Moreover, when treated with heat-inactivated CRE (HI-CRE), wherein proteolytic activity was ablated, cell viability remained unaffected (Figure 3B). None of the protease inhibitors examined adversely affected 16HBE cell viability (Figure 3C). When 16HBE cells were co-treated with CRE and protease inhibitor compounds, PE-BBI significantly rescued cockroach-induced cell damage (*p* < 0.05)—in contrast to pLR-HL and GM, which failed to do so (Figure 3D). 

### 3.4. PE-BBI Prevented CRE-Mediated Epithelial Barrier Disruption 

**Figure 4:**
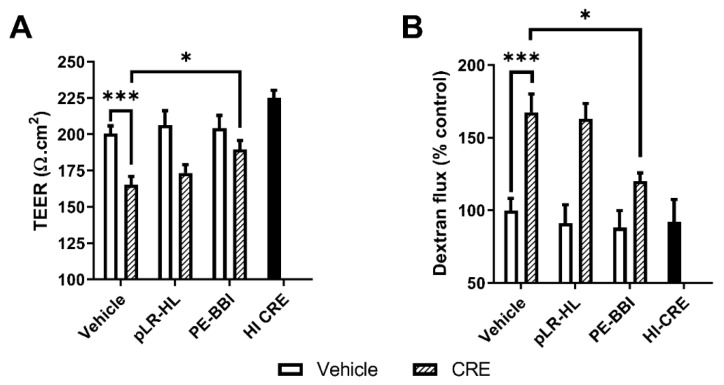
The impact of protease inhibitors on CRE-mediated epithelial barrier disruption. Epithelial barrier function was measured by (**A**) TEER measurement and (**B**) Texas red dextran flux. CRE and heat-inactivated (HI)-CRE were added at the same concentration (16.5 µg protein/well). Data are presented as the mean ± SEMs (n ≥ 4). * P < 0.05, *** P < 0.001.

Epithelial barrier dysfunction, in individuals with asthma, increases susceptibility to environmental agents, including allergens. We found that CRE treatment reduced transepithelial electrical resistance (TEER) values in polarised 16HBE cells by ~25%, an effect that was significantly rescued by PE-BBI (p < 0.05), but not pLR-HL co-treatment (Figure 4A). Consistent with these findings, we also found a significant increase in paracellular flux in CRE-treated cells compared with HI-CRE control (p < 0.05) (Figure 4B). This effect was again reversed by PE-BBI co-treatment (p < 0.05) but not pLR-HL (Figure 4B). 

## 4. Discussion

Proteases from a variety of aeroallergens including cockroach have been implicated in initiating and exacerbating allergic responses, with the airway epithelial barrier representing the first line of defence. To activate the immune cascade, allergens must disrupt the epithelial barrier and gain access to subepithelial immune components such as dendritic cells, ultimately promoting a Th2 phenotype. Normally, the epithelial barrier consists of tight junctions enabling tight cell–cell contacts which act as a physical barrier to macromolecules. However, in allergic asthmatics, an impaired epithelium is evident [23,24]. Previous studies highlight that cockroach proteases increase transepithelial permeability and cytokine production [5,8,12,13,25,26,27,28]. Consequently, inhibition of cockroach TLP activity offers potential therapeutic benefit. The central finding of the current investigation is that a recently identified serine protease inhibitor PE-BBI [14] potently inhibits CRE proteolytic activity, which results in protection of airway epithelial cultures (16HBE) from CRE-induced cell damage and barrier disruption. 

For comparative purposes, we employed GM, alongside our novel compounds, PE-BBI and pLR-HL, as a recent study reported reduced CRE-mediated cell damage and inflammation (BEAS-2B cells) in the presence of this compound [13]. In contrast to the previous report, we did not find that GM significantly affected CRE-mediated loss of cell viability. Notably, different cell types were used in the two studies, with BEAS-2B cultures utilised by Lee and colleagues [13], whereas 16HBE14o^-^ cells were examined in the present study. Furthermore, it is important to highlight the potential for variability between cockroach preparations across the studies. There was a trend towards modest protection of cells by GM, and pLR-HL, but this was not statistically significant. This may be reflective of their more modest potency against CRE cockroach TLP activity than the more active PE-BBI which showed promising benefits in our study. These findings, however, reinforce issues regarding the damaging effects of proteolytic activities contained in CRE on airway epithelium to include barrier disruption. This is further supported by our observation that heat-inactivated CRE, wherein proteolytic activity was ablated, had no effect on airway epithelial cell viability or barrier function. 

Bowman–Birk inhibitors contain a canonical disulphide-bridged loop between cysteine residues, essential for their trypsin inhibitory capability [29]. Comparing the primary structure of PE-BBI and pLR-HL, both have an identical amino acid sequence contained within the same disulphide-bridged loop structure but differ entirely at the N-terminal and C-terminal extension amino acid residues. Interestingly, this suggests that these terminal residues contribute to the potency of these peptides regarding CRE serine protease activity, with PE-BBI greater than a magnitude fold more potent than pLR-HL. It will be of interest to further investigate the structure–activity relationship of the peptides to better understand the underlying importance of these N-terminal and C-terminal extension amino acid residues. 

To date, the identification and characterisation of selective inhibitors of allergen-associated proteases has received little attention with focus predominantly placed on the inhibition of host protease activities [30]. Here, we report that PE-BBI does not significantly affect serine protease activity at the mucosal surface of polarised airway epithelial cultures, in contrast to GM or pLR-HL. Inhibitors such as PE-BBI may therefore represent a valuable tool to help discern the specific role of allergen serine protease activity when tested within complex human cell models. To our knowledge, there are no other serine protease inhibitors that display selectivity for cockroach serine protease activity over host activities. 

As allergen proteases elicit damage of the airway epithelial barrier facilitating allergic sensitisation of the underlying cells and tissues, it seems plausible that inhibition of these protease activities may confer therapeutic benefit. Further work is, however, required to demonstrate whether this is indeed the case. Importantly, consideration must also be given to the potential timing and feasibility of efficacious intervention, as protease allergens are known to play a key role during the initial sensitisation phase. As such, it is still unclear as to whether treatment would have to be prophylactic or whether protease inhibition could provide benefit during acute episodes of allergen protease exposure or perhaps whether treatment could contribute to long-term alleviation of symptoms. 

## 5. Conclusions

PE-BBI represents a novel peptide-based inhibitor that potently and selectively targets CRE TLP over host protease activities, which consequently confers protection against airway epithelial barrier disruption.
